# Pfizer-BioNTech COVID-19 Vaccine Tolerance in Allergic versus Non-Allergic Individuals

**DOI:** 10.3390/vaccines9060553

**Published:** 2021-05-25

**Authors:** Marita Nittner-Marszalska, Marta Rosiek-Biegus, Agnieszka Kopeć, Robert Pawłowicz, Magdalena Kosińska, Aleksandra Łata, Leszek Szenborn

**Affiliations:** 1 Department and Clinic of Internal Medicine, Pneumology and Allergology, Wroclaw Medical University, Str. Marii Skłodowskiej-Curie 66, 50-369 Wrocław, Poland; marita.nittner-marszalska@umed.wroc.pl (M.N.-M.); marta.rosiek-biegus@umed.wroc.pl (M.R.-B.); agnieszka.kopec@umed.wroc.pl (A.K.); magdalena.kosinska@umed.wroc.pl (M.K.); lataaleksandra@gmail.com (A.Ł.); 2 Department of Paediatrics and Infectious Diseases, Wroclaw Medical University, Str. Chałubińskiego 2-2a, 50-368 Wrocław, Poland; leszek.szenborn@umed.wroc.pl

**Keywords:** COVID-19, vaccination, allergic reaction

## Abstract

Individuals with a history of allergy are potentially at risk of suffering from adverse effects after COVID-19 vaccination. We sought to assess the tolerance towards the Pfizer-BioNTech vaccine in allergic patients. To address this issue, we used a questionnaire conducted on-line in a group of medical professionals who were vaccinated with the Pfizer-BioNTech vaccine. A total of 1808 respondents, out of whom 1707 received two doses of the vaccine, returned the questionnaire. Local reactions after injection were more frequent in allergic individuals after both doses (swelling *p* = 0.0003). Systemic adverse events (AE-SYS) occurred more often after the second than the first dose in both groups (allergic persons: 77.29% vs. 41.06%); vomiting and arthralgia occurred more often in allergic subjects (*p* = 0.0009). AE-SYS in allergic individuals lasted longer than in non-allergic ones after the first (*p* = 0.01) and the second dose (*p* = 0.0009). Allergic reactions after vaccination were reported more frequently in allergic subjects: after the first dose (*p* = 0.00001) and after the second dose (*p* = 0.001). Rhinitis was the most frequent symptom observed more often in allergic patients. No severe allergic reactions occurred during the full cycle of vaccination. Although the Pfizer-BioNTech vaccine is tolerated worse by allergic than non-allergic individuals, the occurring adverse symptoms are mild and do not preclude a successful completion of the vaccination cycle. The presence of symptoms suggestive of allergy does not constitute a condition of increased risk of developing clinically significant adverse events following Pfizer COVID-19 vaccination.

## 1. Introduction

Vaccination, one of the most effective prevention instruments against contagious diseases, has affected a near-eradication of smallpox, measles and a successful confinement of the incidence of polio. Nowadays, global vaccination programs provide every prospect of confining and controlling the present SARS-CoV-2 pandemic [1,2]. For a long time now, vaccination has been proven to be a safe medical procedure. Nevertheless, what may cause anxiety is the possibility of the incidence of immune-mediated adverse reactions, including anaphylaxis [3,4,5]. Vaccine-specific rate of anaphylaxis is reported as 1.31 (95% CI 0.90–1.84) per million doses, with no fatalities occurring [6,7,8].

The first COVID-19 vaccine approved by world regulatory agencies to prevent severe acute respiratory syndrome coronavirus 2 (SARS-Cov-2) was the Comirnaty vaccine developed by Pfizer-and BioNTech [9]. Early data on the occurrence of adverse events following a Comirnaty shot prove the vaccine’s safety [10,11,12]. Comirnaty was approved for use in the USA, Canada, and Europe in December 2020 [13,14].

In the USA, the very first doses were recommended for health care personnel and long-term care facility residents. After administering 1.9 mL of the first doses of Pfizer-BioNTech COVID-19 vaccine in the USA, 4393 (0.2%) adverse events were reported, including 83 allergic events and 21 cases of anaphylaxis [15]. Most of the anaphylactic reactions (17/21, 81%) occurred in individuals with documented history of hypersensitivity to a number of allergens, like medications, foods, and stinging insect venoms.

Since over 30% of world population are affected by allergic conditions (the data may be underestimated), in the situation of mass vaccination against COVID-19, it is important to carry out a thorough assessment and resultant stratification of the actual risk of administering the vaccine to allergic patients, not only after the first but also after the second dose [16,17,18]. Of import in collecting such data are reporting systems based of spontaneous reports, like VARES (Vaccine Adverse Event Reporting System) and V-safe system [19,20]. Our study was devised as an assessment of post-vaccination symptoms based on a questionnaire conducted in a numerous group of respondents who received the first and the second dose of the Pfizer vaccine. The aim of the study was to assess course of vaccination procedure in the whole population of medical staff and students of Wrocław Medical University, Poland.

## 2. Materials and Methods

### 2.1. Study Design Material and Methods

The questionnaire-based study was conducted in a group of medical professionals and medical students at Medical University of Wroclaw, Poland, who were vaccinated with Pfizer-BioNTech Comirnaty vaccine between 28 December 2020 and 1 February 2021. On 2 February, when the vaccination cycle was completed and the second doses were administered, the questionnaire comprising 18 items was sent to the potential respondents’ email addresses at Wroclaw Medical University and the University Hospital in Wroclaw. The email contained an invitation to the study and a link to the questionnaire. To create questionnaire, we used Google Forms software included as part of the free, web-based Google Docs Editors suite. The questionnaire was analyzed and assessed by a panel of professionally competent judges (14 physicians and 12 nurses) who evaluated understandability and clarity of the questionnaire items. The final version of the questionnaire was the outcome of their coordinate effort.

### 2.2. Statistical Analysis

All categorized variables are given as numbers and percentages. The statistical significance of differences between the groups were assessed using: Chi2 test. The *p* < 0.05 was considered statistically significant. Statistical analyses were performed using STATISTICA 13 (StatSoft).

## 3. Results

### 3.1. Baseline Demographic and Clinical Characteristics of the Study Group

The size of the studied population was 2600 and response rate was 69%. There were 1707 questionnaires returned by individuals who confirmed they had received two doses of the Pfizer-BioNTech vaccine, which were further analyzed. In the remaining 99 cases, respondents still waited to receive the second dose, and in 2 cases the second dose was postponed due to reasons other than the occurrence of an allergic reaction after the first dose. Among the twice-vaccinated respondents, women and individuals under 50 prevailed. The demographic data of both groups is presented in Table 1.

Among the concomitant diseases reported by the twice-vaccinated individuals (*n* = 1707) were: cardio-vascular diseases (9.43%), asthma (6.21%) and thyroid disease (7.53%). Among the most frequently reported factors indicated as responsible for the occurrences of allergic conditions were inhaled allergens (34.38), food allergens (10.54%), drugs (7.91%), and insect venoms (4.56%). The reported anaphylactic reactions were identified as being triggered by drugs (1.52%), food (1.17%), insect venoms (0.89%), and other factors (0.59%).

### 3.2. Local and Systemic Adverse Events after the First and the Second Dose of Pfizer-BioNTech COVID-19 Vaccine

The frequency of adverse events (all local and/or systemic) after the first dose was 92.03%, and after the second dose 92.97%. The incidence of specific local symptoms did not differ between the first and the second dose, except for pain at the site of injection that was reported to occur more often after the second dose (*p* < 0.01).

Individuals with a history of allergy, as compared to those without one, more often reported local symptoms (swelling, redness), both after the first and the second vaccine dose (*p* < 0.01) (Table 2).

The frequency of systemic adverse events (occurrence of more than one adverse symptom) was higher after the second dose (73.64%) than after the first one (38.37%) in the whole analyzed group as well as in the subgroup of allergic individuals, after the second dose (77.29%) and after the first dose (41.06%). After the first dose, vomiting was more frequently reported in allergic respondents than in non- allergic ones. After the second dose, allergic respondents more often reported arthralgia, headache, gait disturbances, and heart palpitations (Table 2).

In allergic respondents, adverse symptoms lasted longer than in non-allergic ones and limited their living activity in greater measure (Table 3 and Table 4). Allergic respondents had to seek medical intervention nearly twice as often as non-allergic ones (4.83% vs. 2.73%) (Table 3).

### 3.3. Allergic Reactions after the First and the Second Dose of Pfizer-BioNTech COVID-19 Vaccine

Allergic reactions were reported by 38 respondents after the first dose and by 48 respondents after the second dose. The frequency of the reactions did not increase after the second dose. In the subgroup of allergic respondents, the frequency of allergic reactions after the second dose of vaccine did not increase, either, except for rhinitis which was observed more frequently after the second dose (*p* = 0.04).

Allergic symptoms were reported more often by individuals with a history of allergy. As compared with individuals without a history of allergy, at least one allergic symptom occurred 4 times more frequently in allergic respondents after the first dose (*p* = 0.00001) and more than twice more often after the second dose (*p* = 0.001).

Patients with a history of anaphylaxis suffered from post-vaccination adverse events more often than patients with a history of allergic reactions, both after the first dose (15.6%) and after the second dose of the vaccine (15.6%). The scope and profile of post-vaccination allergic reactions were fairly similar in the groups of patients with a history of anaphylaxis and a history of allergic reactions.

## 4. Discussion

The questionnaire-based study was meant to monitor the safety of the two-dose cycle of vaccination with mRNA Pfizer-BioNTech (Comirnaty) and assess the risk of adverse events in individuals with a history of allergy/anaphylaxis as compared with the remaining non-allergic vaccinated population. The study was carried out during the first month of the vaccination program in Poland in a group of medical professionals who were vaccinated first on a high-priority basis.

During the initial, more general stage of the analysis of the gathered material, it could be noticed that occurrences of post-vaccination local and systemic symptoms in the whole study population (regardless of the presence or absence of a history of allergy/anaphylaxis) are not infrequent. The most typical symptoms after the first dose were headache (16.2%), myalgia (18%) arthralgia (8.4%), and fatigue (23.55%). Irrespective of a different mode of acquiring data, our results were close, both qualitatively and quantitatively, to the currently available early reports based on the VARES system involving non-LTCF residents and the v-safe system established specially for the COVID-19 vaccination program to assess the scale problems the endeavor involves [15,21]. The VARES/v-safe data cover observations collected from 12 December 2020 to 13 January 2021 when 13,794,904 COVID-19 vaccine doses were administered in the USA, among these, 80.8% were the Pfizer-BioNTech vaccine [21]. The most frequently reported symptoms after the first dose, according to the v- safe system, were similar to those of our study: headache (17.5%), myalgia (14.7%), and fatigue (21.9%). After the administration of the second dose of Comirnaty vaccine, in large measure the adverse systemic symptoms were reported more frequently in the study population: headache (41.8%), arthralgia (30.5%) and fatigue (49.6%). By comparison, the available USA data after the second dose report similar values: headache (43.4%), arthralgia (23%), and fatigue (53%).

The second stage of the study applied exclusively to vaccinated individuals with a history of allergy/anaphylaxis. While admittedly allergic subjects reported post-vaccination adverse symptoms more frequently (two- to fourfold increase) than non-allergic ones, this tendency largely concerned local reactions. Additionally, after either dose, the symptoms lasted longer in allergic individuals and in greater measure affected their living activity and/or required medical intervention. It is not clear why patients with a history of allergy/anaphylaxis showed non-allergic post-vaccination symptoms (headache, fever, etc.), both local and systemic ones, more frequently than non-allergic individuals. Both these types of reactions are attributed to non-specific inflammation due to the injection itself (injection of foreign materials) which in allergic patients can be excessive. The phenomenon can be attributed to lower reactivity threshold of effector cells (mast cells among them) to non-IgE activating factors as well as to the activation of other cell populations such as macrophage, dendritic cells, eosinophils, basophils and especially innate type 2 lymphoid cells.

In both groups (allergic vs. non-allergic) occurrences of reactions that might be classified as symptoms of hypersensitivity were rare. Additionally, they were rather mild and none of the reactions constituted a contraindication to continuing vaccination. It should be also emphasized that none of the vaccinated persons developed severe anaphylaxis. Considering the actual low percentage and mild severity of the post-vaccination adverse events in patients with a history of allergic reactions, a more detailed analysis of the influence of specific allergies on the occurrence of adverse events was not undertaken. Our primary objective was to estimate the risk of post-vaccination allergic reactions in patients with a history of anaphylaxis, a history of allergic reactions and/or a history of episodes that might be classified as hypersensitivity ones. The study results indicate that these persons may be at a heightened risk of post-vaccination adverse events, though of mild severity. Similar conclusions can be drawn from the data on the occurrence of allergic adverse event recorded in the VARES system. Concerning systemic allergic reactions after the first dose of the vaccine, 17/21 patients have a history of allergies caused by drugs, insect venoms, food allergens, or vaccines.

After the second vaccine dose, the reporting of hypersensitivity symptoms did not increase, with the exception of rhinitis that was reported twice as frequently as after the first dose. As prescribed by the Pfizer-Biontech vaccination protocol, two doses of the vaccine have to be administered to achieve the expected immunization effect. Sensitization could occur during the administration of the first dose, which might result in the vaccinated individuals developing allergic reactions to the second dose. The results of our study indicate that neither in the patients with a history of allergy/anaphylaxis nor in the non-allergic group, administration of the first dose effected sensitization to the vaccine components that would result in a higher rate of hypersensitivity reactions after the second dose. It needs to be emphasized, however, that the study patients with a history of allergy and/or anaphylaxis who demonstrated good vaccine tolerance constituted a group of individuals who had been qualified for vaccination following guidelines and recommendations prerequiring stratification and phenotyping of such patients [22,23]. Patients with a history of anaphylaxis to vaccines and injection drugs containing polyethylene glycol (PEG), as potentially susceptible to hypersensitivity reactions to the PEG component of vaccines [24,25,26], had undergone allergological consultations recommended by the Polish Allergological Society prior to being vaccinated [27,28,29,30].

We are aware that the findings presented in this study may have some limitations. Admittedly, questionnaire based studies are subject to subjective responses about the reported symptoms and so reporting biases are possible, both resulting from overreporting arising from increased awareness and from underreporting arising from lack of awareness of the survey requirements. The above considered, the percentage of persons reporting allergies is likely to be overestimated, an example being food allergy reported 5 times more often in our survey than in general adult population. Here, the misreporting can be accounted for by the fact that many patients are sincerely convinced they are allergic to foods without being professionally diagnosed, often attributing their presumed condition to adverse symptoms that merely testify to food intolerance. However, with regard to the reporting of inhalant allergy (34%), drug allergy (8%), and hymenoptera venom allergy (4.6%), the data do not diverge from general epidemiological statistics. Moreover, some of the patients reported more than one allergy triggering factor, which lowered the percentage of allergic individuals in the study population. Finally, it is a matter of fact that the adverse reactions reported after vaccination were undeniably based on subjective estimates by the surveyed study group members. However, in the case of our study, which was conducted on medical professionals, it can be expected that the responses should be more objective. And to further mitigate the objection of lack of objectivism of questionnaire-based studies, we want to observe that in situations of real-life qualification for anti-COVID-19 vaccination, medical personnel have to rely on an initial anamnesis for the administration of the first dose and on the already vaccinated patient’s verbal statement for the second one.

## 5. Conclusions

In conclusion, the findings of our study indicate that vaccination with Pfizer-BioNTech anti COVID-19 vaccine is well tolerated by both persons with and without a history of allergy/anaphylaxis. The vast majority of the reported symptoms are local. The character and intensity of the reactions do not represent an obstacle to a successful completion of the vaccination cycle. The presence of symptoms seemingly suggestive of allergy (but not diagnostically confirmed as being of allergic character) does not constitute a condition of increased risk of developing clinically significant adverse events following Pfizer COVID-19 vaccination.

## Figures and Tables

**Table 1:** Baseline demographic and clinical characteristics of the study group.

Variable	Respondents Vaccinated with the First Dose of Comirnaty	Only Respondents Vaccinated with Both the First and the Second Dose of Comirnaty
Number of returned questionnaires	1808	1707
Male, *n* (%)	375 (20.74%)	356 (20.85%)
Age (years)		
20–29	606 (33.52%)	585 (34.27%)
30–39	595 (32.91%)	554 (32.45%)
40–49	281 (15.54%)	264 (15.47%)
50–59	171 (9.46%)	160 (9.37%)
>60	155 (8.57%)	144 (8.44%)
Allergies or allergic reactions in the past	879 (48.62%)	828 (48.51%)
Previous anaphylaxis episode	70 (3.87%)	64 (3.75%)
Concomitant diseases	673 (37.22%)	633 (37.08%)

**Table 2:** Characteristics of reported adverse symptoms after receipt of the first and the second dose of Comirnaty vaccine in the study group (*n* = 1707).

**Symptoms after Vaccination**	Allergic * Respondents	**Non-Allergic Respondents**	***p***
After the first dose of the vaccine			
Fever (above 38 °C)	13(1.6%)	12 (1.4%)	0.73
Low grade fever	83 (10%)	75 (8.5%)	0.29
Arthralgia	80 (9.7%)	65 (7.4%)	0.09
Myalgia	155 (18.7%)	153 (17.4%)	0.48
Headache	149 (18%)	129 (14.7%)	0.06
Palpitations	19 (2.3%)	15 (1.7%)	0.38
Vomiting	10 (1.2%)	3 (0.3%)	0.04
Gait disturbances **	6 (0.7%)	3 (0.3%)	0.27
Other cardiovascular symptoms	1 (0.1%)	4 (0.5%)	0.2
Other respiratory symptoms	1 (0.1%)	2 (0.2%)	0.59
Other neurological symptoms	15 (1.8%)	7 (0.8%)	0.06
Local swelling	158 (19.1%)	111 (12.6%)	0.0003
Local redness	144 (17.4%)	98 (11.2%)	0.0002
Local pain	714 (86.2%)	699 (79.5%)	0.0002
Loss of smell and/or taste	2 (0.2%)	3 (0.3%)	0.7
**After the Second Dose of the Vaccine**	**Allergic Respondents**	**Non-Allergic Respondents**	***p***
Fever (above 38 °C)	142 (16.2%)	122 (13.1%)	0.07
Low grade fever	245 (27.9%)	250 (26.9%)	0.65
Arthralgia	279 (31.7%)	243 (26.2%)	0.009
Myalgia	421 (47.9%)	405 (43.6%)	0.07
Headaches	368 (41.9%)	346 (37.2%)	0.04
Palpitation	57 (6.5%)	38 (4.1%)	0.02
Vomiting	24 (2.7%)	10 (1.1%)	0.009
Gait disturbances **	18 (2.1%)	4 (0.4%)	0.002
Other cardiovascular symptoms	15 (1.7%)	5 (0.5%)	0.02
Other respiratory symptoms	7 (0.8%)	5 (0.5%)	0.49
Local swelling	159 (18.1%)	112 (12.1%)	0.0003
Local redness	123 (14%)	99 (10.7%)	0.03
Local pain	643 (70.2%)	657 (70.7%)	0.25
Loss of smell and/or taste	3 (0.3%)	3 (0.3%)	0.95
Fatigue	436 (49.6%)	416 (44.8%)	0.04

* Allergic respondents e.g., reporting allergy and/or anaphylaxis in the past ** Gait disturbances and dysesthesia in the limbs.

**Table 3:** Impact of adverse symptoms on the living activity of individuals receiving Pfizer-BioNTech anti COVID-19 vaccine.

**First Dose**	**Allergic Respondents**	**Non-Allergic Respondents**	***p***
No negative impact	354 (42.75%)	408 (46.47%)	0.006
Low negative impact	344 (41.55%)	325 (37.02%)	
Pharmacological intervention was required.	70 (8.45%)	58 (6.61%)	
Living activity considerably limited. Medical consultation was required.	3 (0.36%)	3 (0.34%)	
**Second Dose**	**Allergic Respondents**	**Non-Allergic Respondents**	***p***
No negative impact	192 (23.19%)	227 (25.82%)	0.02
Low negative impact	278 (33.57%)	287 (33.65%)	
Pharmacological intervention was required.	285 (34.42%)	282 (32.08%)	
Living activity considerably limited. Medical consultation was required.	40 (4.83%)	24 (2.73%)	

**Table 4:** Duration of systemic adverse symptoms in individuals receiving Pfizer-BioNTech anti COVID-19 vaccine.

**After the First Dose Adverse Systemic Reactions Persisted:**	**Allergic Respondents**	**Non-Allergic Respondents**	***p***
<24 h	173 (20.89%)	209 (23.78%)	0.01
24–72 h	524 (63.29%)	533 (60.64%)	
>72 h<7 days	60 (7.25%)	39 (4.44%)	
>7 days	8 (0.97%)	5 (0.57%)	
**After the Second Dose Adverse Systemic Reactions Persisted:**	**Allergic Respondents**	**Non-Allergic Respondents**	***p***
<24 h	191 (23.07%)	230 (26.17%)	0.0009
24–72 h	522 (63.04%)	540 (61.43%)	
>72 h<7 days	55 (6.64%)	35 (3.98%)	
>7 days	13 (1.57%)	2 (0.23%)	

## Data Availability

The data presented in this study are available on request from the corresponding author.
